# Elevated Alanine Aminotransferase Is Strongly Associated with Incident Metabolic Syndrome: A Meta-Analysis of Prospective Studies

**DOI:** 10.1371/journal.pone.0080596

**Published:** 2013-12-04

**Authors:** Zhengtao Liu, Shuping Que, Huaijun Ning, Linlin Wang, Tao Peng

**Affiliations:** 1 Department of Hepatobiliary Surgery, First Affiliated Hospital of Guangxi Medical University, Nanning, Guangxi Province, China; 2 Department of Pediatrics, Women and children’s hospital of Guangxi, Nanning, Guangxi Province, China; 3 Department of Pediatrics, First Affiliated Hospital of Guangxi Medical University, Nanning, Guangxi Province, China; University of Sao Paulo, Brazil

## Abstract

**Background:**

The incidence of metabolic syndrome (MetS) is rapidly increasing worldwide and associated with alanine aminotransferase (ALT) activity. However, the impact of ALT activity on MetS incidence is inconsistent in published literature. We therefore estimated the association between elevated ALT activity and incident MetS through a meta-analysis of prospective cohort studies.

**Methods/Principal Findings:**

All published prospective cohort studies on the association between elevated ALT activity and incident MetS were retrieved from Pubmed, Embase, and the Institute for Scientific Information (ISI). In all, seven prospective cohort studies, with 31545 participants and 2873 cases of incident MetS were recruited. If there was insignificant heterogeneity (P-value>0.05 and I^2^<50%), the fixed-effect model was used to calculate the pooled relative risks (RRs) of incident MetS induced by raised ALT. Otherwise, the random-effect model was used. The calculated RR was 1.81 (95% confidence interval [CI]: 1.49–2.14) when the incidence of MetS was compared between the highest versus the lowest classification of ALT activities. The pooled RR was 1.13 (95% CI: 1.11–1.16) in dose-response analysis with 5 units per liter (U/l) of ALT increment. Subgroup analysis suggested that gender disparity might be the main origin of heterogeneity in overall analysis (P = 0.007 between RRs of gender-specific subgroups evaluated with 5 U/l increments of ALT). Women had a higher dose-response risk of MetS incidence (1.38, 95% CI: 1.20–1.55) than men. Furthermore, sensitivity analysis confirmed the stability of results. No publication bias was found in our meta-analysis.

**Conclusions/Significance:**

Current evidence from prospective studies supports the association between ALT elevation and increasing MetS incidence. This association is closer and more consistent in female population. Further studies are needed to confirm this association and to investigate the potential mechanism of ALT activity on MetS occurrence.

## Introduction

Metabolic syndrome (MetS) is a constellation of metabolic disorders including glucose intolerance, central obesity, dyslipidaemia and hypertension [1]. In epidemiological surveys, MetS is associated with increased risk of incident cardiovascular diseases (CVD) [2], and diabetes [3]. Individuals with MetS had approximately a 45% higher all-cause mortality and a 78% higher coronary heart disease-induced deaths compared to individuals without MetS in previous meta-analyses [4], [5]. Recently, MetS has caused a global epidemiological concern followed with the increasing prevalence of obesity [6] and sedentary behavior [7]. In addition, MetS was also found to be associated with insulin resistance in liver [8].

Alanine aminotransferase (ALT) is routinely measured in a useful screening assay for the detection of non-alcoholic fatty liver disease (NAFLD) in the general population [9]. NAFLD, a manifestation of MetS in liver, is reportedly associated with insulin resistance [10]. Accordingly, ALT activity is associated with MetS through NAFLD. Several prospective studies have therefore been carried out to address the association between ALT activity and the risk of incident MetS in the general population [11]–[17]. However, the results have been inconsistent in the literature due to possible variations including ethnicity, gender, and the selection criteria for participants. Therefore, a meta-analysis was performed on several longitudinal studies to quantitatively evaluate the prospective association between ALT activity and incident MetS. A dose-response analysis was conducted on relative risks (RRs) of incident MetS and individual MetS components (including hyperglycemia, abdominal obesity, hypertriglyceridaemia, low high-density lipoprotein cholesterol [HDL-C] and hypertension) caused by 5 units per liter (U/l) of ALT increments. Furthermore, we performed a subgroup analysis to assess the effects of potential confounders that might cause heterogeneity in meta-analysis. To the extent of our knowledge, this is the first meta-analysis to assess the risk of MetS incidence caused by ALT elevation.

## Methods

### PRISMA and Flow Diagram

Meta-analysis was conducted according to the guidelines of the Preferred Reporting Items for Systematic Reviews and Meta-Analyses (PRISMA) [18]. The supporting PRISMA checklist and flow diagram for this meta-analysis is available as supporting information; see Checklist S1 and Flow Diagram S1, respectively.

### Search Strategy, Study Selection and Data Extraction

Extensive literature retrieval was performed in Pubmed, Embase and the All Database of Institute for Scientific Information (ISI) without language restrictions (last updated at Aug 10, 2013). The search was restricted to studies conducted in human subjects. The medical subject headings “alanine aminotransferase”, “ALT”, and “metabolic syndrome” were used for searching the relevant literature. The following search strategy was applied in Pubmed: (alanine aminotransferase [MeSH] OR alanine aminotransferase [All Fields] OR ALT [All Fields]) AND (metabolic syndrome [MeSH] OR metabolic syndrome [All Fields]). A similar strategy was applied to Embase and ISI. In addition, a manual search of citations from relevant original studies and review articles was performed. Our meta-analysis was performed in accordance with the Meta-analysis Of Observational Studies in Epidemiology (MOOSE) guidelines [19]. Only reports fulfilling the following inclusion criteria were recruited in the meta-analysis: 1) Prospective cohort studies were published as original articles; 2) ALT baseline activities were reported and MetS was an end point; 3) participants with MetS at baseline were excluded completely; and 4) Studies that contained the RR of incident MetS associated with ALT activity and the corresponding 95% confidence interval (CI) were provided or obtained by calculation.

Two authors independently extracted the information using a standardized protocol and reporting form. The agreement was measured using Cohen’s Kappa [20] and discrepancies were resolved by discussion and consensus. Study characteristics were recorded as: first author, publication year, country of origin, and clinical characteristics (numbers of participants/cases, gender category, age range of populations, statistics of comparison, durations of follow-up, diagnostic criteria of MetS, the most fully adjusted RRs of MetS, individual MetS component estimated from multivariable analysis, and study-specific adjusted covariates). When effects estimated in the same population were reported in different follow-up durations, only data with the longest follow-up duration were included.

### Quality Assessment

The quality of each study was assessed through two authors independently using the Newcastle-Ottawa Scale (NOS) [21]. The NOS consists of three parameters of quality: selection, comparability, and outcome for cohort studies (Table 1). The NOS assigned a maximum of four stars for selection, two stars for comparability, and three stars for outcome; therefore, nine stars reflected the highest quality. Studies with >6 stars were considered high in quality. When discrepancies occurred, a joint revaluation of the original article was performed with a third author.

**Table 1:** Check List for Quality Assessment and Scoring of Nonrandomized Studies.

Check List
Selection
1. How representative was the selected group in comparison with the general community population? (if yes, one star; no star if the participants were selected or selection of group was not described)
2. How representative was the group with elevated ALT in comparison with the group within normal range? (if drawn from the same community, one star; no star if drawn from a different source or selection of group was not described)
3. Ascertainment of high risk group in exposure of high ALT activity (if yes, one star)
4. Demonstration that the MetS outcome was not present at start of study (if yes, one star)
Comparability
5. Comparison was controlled for age and gender (if yes, one star; no star was assigned if the two groups differed)
6. Comparison was controlled for alcohol intake, cigarette smoking, family history (one star was assigned as if two or more of these three characteristics were controlled for; no star was assigned if one or less characteristic was controlled for)
Outcome assessment
7. Clearly defined MetS outcome by certain criteria (yes, one star for information ascertained in literature; no star if this information was not reported)
8. Adequate duration of follow-up for observation of ensuing MetS outcome (one star if duration of follow-up>5 year)
9. Adequacy of follow-up of cohorts (one star if follow-up rate >90%)

Abbreviations: ALT: Alanine aminotransferase; MetS: metabolic syndrome.

### Statistical Analysis

RR was chosen to estimate the association between ALT and incident MetS. Heterogeneity between studies was evaluated by the Chi-square-based Q test and I^2^ test with the use of Stata metan [22]. If there was no significant heterogeneity (P-value >0.05 and I^2^<50%), the fixed-effect model will be chosen to estimate the overall RR and 95% CI. Otherwise, the random-effect model will be used. First, combined RRs of MetS were calculated between the highest and lowest categories of ALT activities adjusted for the most potentially confounding covariates (overall and stratified by diagnostic criteria). In addition, the dose-response association between the risks of incident MetS per 5 U/l increment of ALT activity was estimated (overall and stratified by diagnostic criteria) according to reported data (categories of ALT activities on median dose, number of cases and participants, and effect estimation with corresponding standard errors) using a method previously proposed [23], [24]. Median or mean values in data of different studies were presented according to the different categories of ALT activities. When this information was unreported, the midpoints of the upper and lower boundaries were assigned as the approximate medians. When the highest category was open-ended, the lower end value of the category multiplied by 1.2 was assigned [25]. In addition, the combined RRs per standard deviation (SD) increment of logALT activity in two studies [12], [15] were analyzed. Meanwhile, pooled RRs of detailed incident individual MetS components per SD increment of logALT activity were evaluated based on data from two studies [12], [15]. Furthermore, analysis was performed in subgroups classified by gender, mean age (the midpoint of the upper and lower boundaries of age when not given in literature), race/ethnicity, follow-up duration, sample size of study population, number of adjusted covariates, difference of selection criteria (including whether diabetics were excluded, and whether diabetics were deliberately included), adjustment of gamma-glutamyltransferase (GGT) or alcohol intake as covariates. The degree of inconsistency among studies (I^2^) was estimated through scores of 25, 50, and 75%, representing low, moderate, or high inconsistency respectively [26].

Finally, we performed a sensitivity analysis to evaluate the influence of a single study on the overall effect estimate by omitting one study at a time with metainf [27]. Begg’s funnel plot and Egger’s test were used to evaluate publication bias with metabias [28]. A P-value <0.05 was considered as significant. The meta-analysis including metan, metainf, and metabias command was performed by Stata 12.0 software (Stata Corp, College Station, TX, USA).

## Results

### Literature Retrieval

**Figure 1:**
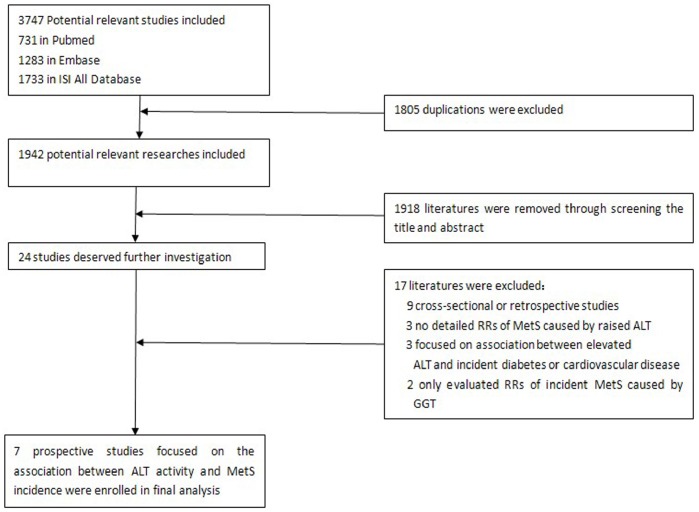
Flow chart of selected studies for meta-analysis.

The flow chart of enrolled studies for meta-analysis is shown in Figure 1. A total of 1942 studies were retrieved from three databases (Pubmed, Embase and the ISI All Database) after exclusion of 1805 duplications. Among the 1942 studies, 1918 were removed by screening the titles and abstracts. The remaining 24 publications were independently screened through two authors (Zhengtao Liu and Shuping Que). Seven papers were finally included (Cohen’s Kappa = 0.755). The process and reasons for exclusion are shown in Figure 1.

### Characteristics of the Enrolled Studies

Seven prospective cohort studies, involving 31545 participants and 2873 cases of incident MetS, were recruited in the meta-analysis. Characteristics from the seven studies are presented in Table 2. Among the seven studies, three were performed in East Asians, two were performed in Americans, and two were performed in Europeans. All of the enrolled subjects were excluded for MetS at baseline and the prevalence of MetS incidence varied from 2.32% to 26.4%. The mean age of participants ranged from 30 to 75 years old. The mean length of follow-up ranged from 3 to 20 years. Among the enrolled studies, one study excluded subjects with excessive alcohol consumption and two studies excluded diabetics. Another study deliberately included a few diabetics (less than 4%) to avoid bias.

**Table 2:** Characteristics of seven cohort prospective studies enrolled in meta-analysis.

First author andpublication year	Country	Enrolled studypopulation (case*/total, baselinecharacteristics)	Gender(female/male)	Age (range,mean±SD)	Comparison(U/L)	Follow-up(years)	Definition ofMetS	RR (95% CI) ofdeveloping MetS	Adjustedcovariates
Nakanishi et al.[11], 2004	Japan	608/2957 withoutMetS	0/2957	39–59	Highest quintile vs.lowest quintile (≥29vs. <14)	7	NCEP ATPIII	1.42 (0.95–2.11)	Age, alcohol intake, cigarettesmoking, family history, regularphysical activity, WBC count,GGT, AST, and ALP
Hanley et al.[12], 2005	USA	127/633 without MetSand diabetics	354/279	40–69	Highest quartile vs.lowest quartile of ALT(data not on shown)	5.2 (4.5–6.6)	NCEP ATPIII	2.12 (1.10–4.19)	Age, sex, ethnicity, clinicalcenter,alcohol intake,waistcircumference, IGT, Si, and AIR
					Per SD increment oflogALT level			1.31 (1.04–1.66)	
Andre et al.[13], 2007	France	309/3545 withoutMetS	1889/1656	30–65 M:46.2±10.0 F: 46.4±9.9	Highest quartile vs.lowest quartile (M:<19.5 vs. ≥33.4; F: <13.5vs. ≥21.3)	3	IDF	M:1.25 (0.70–2.21)F:2.87 (1.40–4.43)	Age, alcohol intake, physicalactivity, smoking habits, andGGT
Schindhelm et al.[14], 2007	Netherlands	226/1097 without MetSand diabetics	632/465	50–75 (60±6.7)	Highest tertile vs.lowest tertile (<12.5vs.≥17.2)	6.4 (4.4–8.1)	NCEP ATPIII	2.25 (1.50–3.37)	Age, sex, alcohol intake, andfollow-up duration
Goessling et al.[15], 2008	USA	621/2557 without MetSand alcohol abuser	1431/1126	44±10	Per SD increment oflogALT level	20	NCEP ATPIII	1.21 (1.09–1.34)	Age, sex, smoking, menopausalstatus, alcohol use (g/day), andinterim weight change
Jo et al.[16], 2009	Korea	M:656/15249 F:146/6286Without MetS butincluding a few diabetics(less than 4%) toavoid bias	6286/15249	M: 37.7±6.50 F:41.5±6.28	Highest quartile vs.lowest quartile of ALT(<19.5 vs.>33.4)	4	IDF AHA/NHLB	M(IDF):3.65 (2.68–4.97)F(IDF):2.11 (1.24–3.58)M(AHA/NHL): 6.16(3.22–11.8) F(AHA/NHLB): 2.06 (1.30–3.25)	Age and GGT
Xu et al.[17], 2011	China	180/681 WithoutMetS	410/271	Non-MetS:61.6±9.2; MetS:61.7±9.6	Highest quartile vs.lowest quartile of ALT33(27–44)vs. 9(7–10)	3.5	NCEP ATPIII	1.38 (0.75–2.53)	Age, sex, occupation, educationallevel, family history of diabetes,smoking status, drinkingstatus, leisure-time activity, BMI,HOMA-IR, and GGT

* represented the number of MetS occurrence in prospective studies.

Abbreviations: AHA/NHLB: American Heart Association/National Heart, Lung, and Blood Institute; AIR: Acute insulin response; ALP: Alkaline phosphatase; ALT: Alanine aminotransferase; AST: Aspartate aminotransferase; BMI: Body mass index; F: Female; GGT: Gamma-glutamyltransferase; HOMA-IR: Homeostasis model assessment index of insulin resistance; IDF: International Diabetes Federation; IGT: Impaired glucose tolerance; M: Male; MetS: Metabolic syndrome; NCEP-ATPIII: The Adult Treatment Panel III of the National Cholesterol Education Program; SD: Standard deviation; S_i_: Insulin sensitivity; VS.: Versus; WBC: White blood cell.

Regarding the diagnostic criteria of MetS, five studies adopted the definition provided by the Adult Treatment Panel III of the National Cholesterol Education Program (NCEP ATPIII) [29], one study applied the definition provided by the International Diabetes Federation (IDF) [30] definition and one study applied both IDF and the American Heart Association/National Heart, Lung, and Blood Institute (AHA/NHLBI) [31] criteria, respectively. For low heterogeneity presented according to different diagnostic criteria in the Korean study [16] (I^2^ = 18.5%, P = 0.268 for men, I^2^ = 0.0%, P = 0.949 for women, data not on shown), the IDF definition was chosen as representative criteria in subsequent evaluation. All but one Japanese study (only focused on men) included both men and women participants.

**Figure 2:**
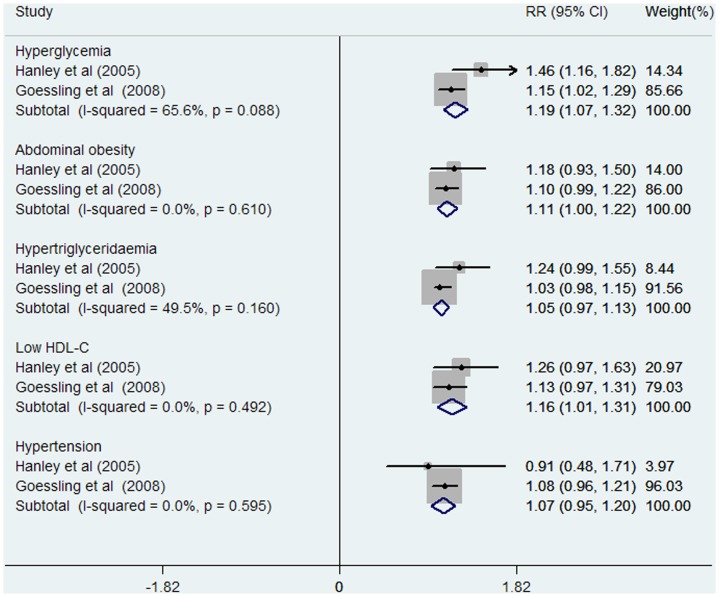
Relative risks of incident individual MetS components per SD increment of logALT. Abbreviations: HDL-C: high-density lipoprotein cholesterol; SD: standard deviation.

Nine data clusters were included in seven studies (two studies provided separate results for males and females). Among them, five studies offered the RRs and 95% CIs of MetS incidence compared between the highest and lowest category of ALT levels. One study reported RR and 95% CI of incident MetS per SD increment of logALT. In addition, one study reported RR and 95% CI of incident MetS between the highest versus lowest category of ALT activity and per SD increment of logALT. Most enrolled studies adjusted for alcohol intake as a covariate, except the Korean study. Detailed RRs of incident individual MetS components (both defined by NCEP ATPIII criteria) per SD increment of logALT are shown in Figure 2.

### Quality Assessment Results

Six of the seven prospective studies were of high quality (NOS score >6). The average NOS score of the studies overall was 7.57. The results of the quality assessment score by NOS are shown in Table S1.

### Quantitative Analysis

**Figure 3:**
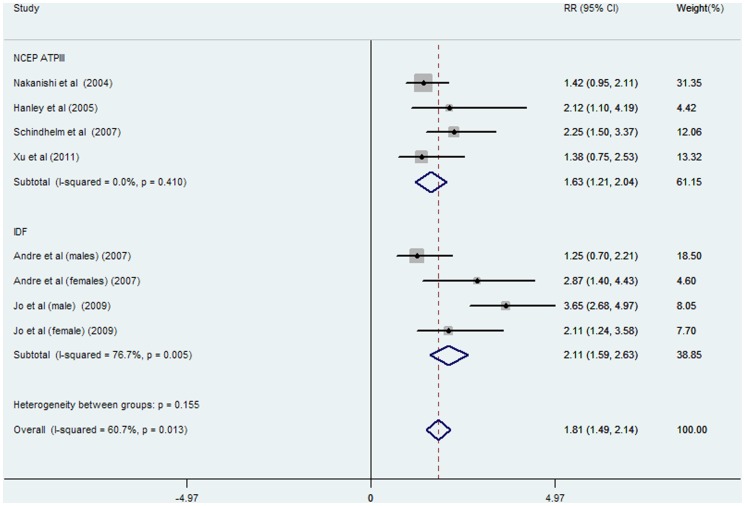
Meta-analysis of comparing relative risk of MetS between the highest versus lowest category of ALT levels classified by different diagnostic criteria. Abbreviations: NCEP-ATPIII: The Adult Treatment Panel III of the National Cholesterol Education Program; IDF: International Diabetes Federation.

The pooled RR of incident MetS was 1.81 (95% CI: 1.49–2.14; P = 0.013, Figure 3) for the highest versus lowest category of ALT activities in eight data clusters from six studies (involving 28988 participants and 1936 cases); high heterogeneity was found in overall (I^2^ = 60.7%, P = 0.013) and IDF-defined studies (I^2^ = 76.7%, P = 0.005) while no statistical heterogeneity was observed in NCEP-ATPIII-defined studies (I^2^ = 0.0%, P = 0.410).

**Figure 4:**
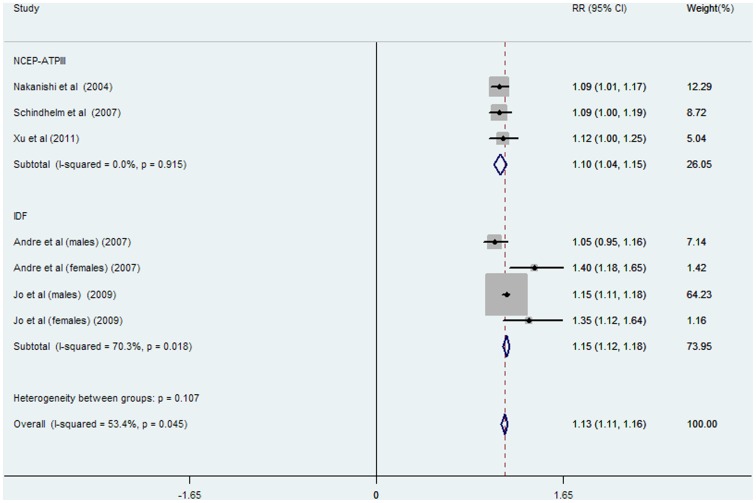
Meta-analysis of comparing relative risk of MetS with 5 U/l of ALT increment classified by different diagnostic criteria. Abbreviations: NCEP-ATPIII: The Adult Treatment Panel III of the National Cholesterol Education Program; IDF: International Diabetes Federation.

RRs and 95% CIs of incident MetS per 5 U/l of ALT increment were reported or calculated in seven data clusters from five studies (involving 28355 participants and 2125 cases, Figure 4). The overall risk of incident MetS increased by 13% (RR: 1.13; 95% CI: 1.11–1.16) per 5 U/l of ALT increment with significant heterogeneity (I^2^ = 53.4%, P = 0.045). High heterogeneity was observed in the IDF-defined subgroup (I^2^ = 70.3%, P = 0.018) but not in NCEP-ATPIII-defined studies (I^2^ = 0%, P = 0.915). No significant heterogeneity was observed between subgroups defined MetS by NCEP ATPIII or IDF criteria in various comparisons (P = 0.155 in Figure 2, and P = 0.107 in Figure 4, respectively).

In addition, the pooled RRs of incident MetS per one SD increment of logALT was exclusively analyzed in two studies [12], [15] (involving 3190 participants and 748 cases). The precise RR was 1.24 (95% CI: 1.10–1.37) with low heterogeneity (I^2^ = 0%, P = 0.606, Figure S1). The pooled RRs of incident individual MetS components caused per SD elevation of logALT were also evaluated in the two studies [12], [15] (Figure 4). The incident hyperglycemia was the only potential individual MetS component outcome in both studies associated with ALT increment with high-pooled RR (1.19, 95% CI: 1.07–1.32) per SD increment of logALT. In addition, no prominent effect of ALT elevation was observed on the other individual MetS component outcome.

### Subgroup and Sensitivity Analyses

**Figure 5:**
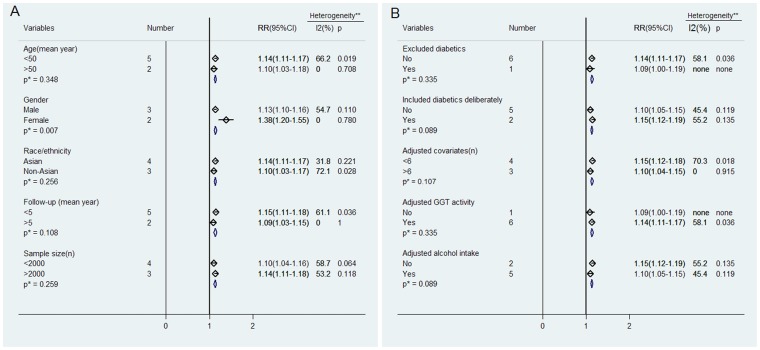
Subgroup analysis on risk of MetS incidence followed with 5 U/l of ALT increment. Abbreviations: GGT: Gamma-glutamyltransferase.

Because heterogeneity was present in overall analysis, we attempted to evaluate the potential source of heterogeneity by subgroup analyses. The effects of ALT increments on the risk of MetS incidence in subgroups are shown in Figure 5. In subgroup analysis, a consistent association between ALT increments and MetS incidence was still observed except in the subgroups classified by gender. In gender-specific subgroup analysis, the dose-response RR of MetS incidence per 5 U/l of ALT increment was significantly higher (RR: 1.38, 95% CI: 1.20–1.55) in female populations with lower heterogeneity (I^2^ = 0.0%, P = 0.780). Otherwise, heterogeneity was significantly attenuated in studies with long-term follow-up (>5 years), more covariates adjusted (n≥6), older (mean age>48 years), and Asian populations. Significant effects on incident MetS caused by ALT elevation were observed in subgroup analysis even after adjusting for alcohol intake and GGT level as covariates.

**Figure 6:**
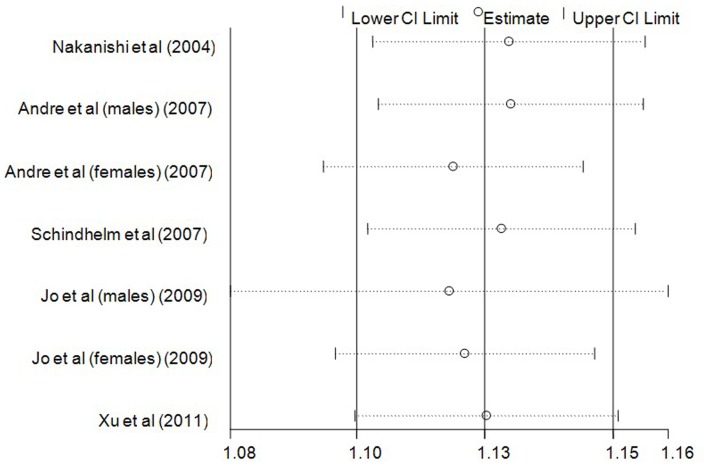
Sensitivity analyses by stepwise omitting one study at a time.

In addition, we conducted sensitivity analyses by stepwise omitting one study at a time and re-evaluated the summary RRs of incident MetS per 5 U/l of ALT increment for the remaining studies to estimate the impact of single studies on combined results. As shown in Figure 6, no single study significantly influenced the pooled RRs.

### Assessment of Publication Bias

**Figure 7:**
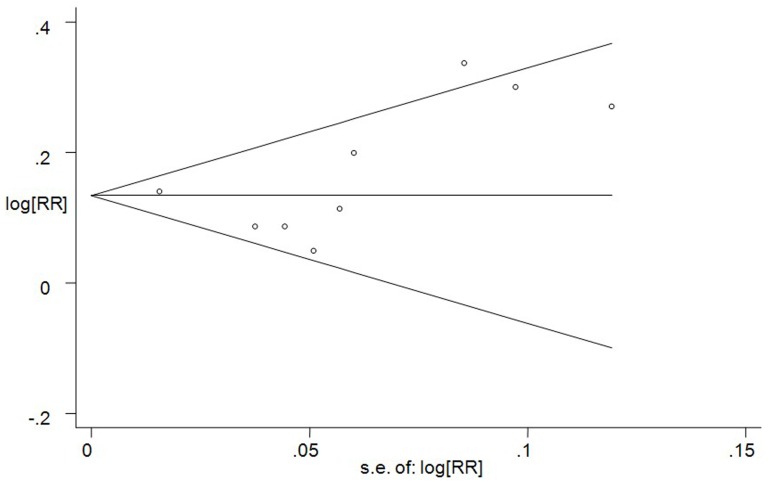
Begg’s funnel plot analysis of publication bias. Egger’s test: P = 0.402.

To assess the publication bias, Begg’s funnel plot (Figure 7) and Egger’s test were used. No significant publication bias were observed (Egger’s P = 0.402).

## Discussion

MetS is a series of metabolic disorders including impaired glucose tolerance, central obesity, dyslipidaemia, and hypertension [1] that was first recognized with an international definition in 1998 [32]. In epidemiological survey, elevated ALT is a marker representing for NAFLD [33], a feature of MetS in liver [8]. Accordingly, many prospective studies were conducted to explore the association between ALT activity and incident MetS in the general population [11]–[17]. In our meta-analysis, seven prospective cohort studies including 31545 participants and 2873 cases of MetS, revealed a significant association between ALT activity and risk of MetS in comparisons between the highest versus the lowest category of ALT activity (RR: 1.81, 95% CI: 1.49–2.14, Figure 3). Dose-response analysis also showed that the risk of MetS incidence was significantly increased with 5 U/l of ALT increment (RR: 1.13, 95% CI: 1.11–1.16, Figure 4). Furthermore, the combined RR of incident MetS was also elevated per SD increment of logALT (RR: 1.24, 95% CI: 1.10–1.37, Figure S1). A high risk of incident hyperglycemia (as an individual MetS component) per SD increment of logALT was observed in both relative studies [12], [15] (RR: 1.19, 95% CI: 1.07–1.32, Figure 2). Subgroup analysis was performed to assess the significant heterogeneity observed in overall analysis. Gender disparity was the remarkable source of heterogeneity between ALT activity and MetS incidence (P = 0.007, Figure 5). The RR of MetS incidence with the same extent of ALT elevation in females (1.38, 95% CI: 1.20–1.55, Figure 5) was significantly and consistently higher (I^2^ = 0, P = 0.780, Figure 5). ALT elevation seems to be a more reliable marker in screening incident MetS in the female population. To the extent of our knowledge, this is the first meta-analysis focusing on the association between ALT activity and ensuing MetS incidence.

Significant heterogeneity was observed in the MetS subgroup defined by IDF criteria in our meta-analysis (Figure 3, Figure 4). However, the source of heterogeneity was more likely from the male data clusters of the enrolled studies [13], [16]. In supporting of our speculation, the male population had higher heterogeneity (I^2^ = 68.1%, P = 0.077, data not shown) than the female population (I^2^ = 0%, P = 0.780, Figure 5) in two studies [13], [16] defined MetS by IDF criteria suggesting that gender rather than diagnostic criteria, is the source of heterogeneity in ALT-MetS incidence association. Accordingly, ALT might be predictive of MetS occurrence despite a NCEP-ATPIII or IDF definition with similar pooled dose-response RRs (Figure 4). However, further studies specifically addressing this notion are needed.

Except for gender, many potential confounders might also be contributing to heterogeneity in the association between ALT activity and MetS incidence. Of them, common factors including alcohol abuse, viral hepatitis, diabetes, and NAFLD status [34] (that may cause ALT elevation at baseline) were not excluded during enrollments of some studies, which might induce heterogeneity in meta-analysis. Therefore, it is necessary to consider the confounders in the enrollment of study participants. With respect to alcohol abuse [35], [36], one study [15] evaluated the RR of MetS incidence caused by ALT elevation, excluding subjects with heavy alcohol intake. Interestingly, statistically low heterogeneity (I^2^ = 0%, P = 0.606; Figure S1) was observed in pooled analyses when combining the studies with or without exclusion of alcohol abusers, indicating that the influence of enrolling alcohol abusers was limited on the ALT-MetS association in the general population.

For diabetes as another potential cause of ALT elevation [35], [37], two studies [12], [14] excluded diabetics at baseline and another study [16] included the diabetics deliberately to avoid bias. Subgroup analysis also showed no significant difference among subgroups that deliberately included, did not exclude and completely excluded diabetics (Figure 5). We speculate that the possible explanation is the low prevalence of diabetics related to the whole population [38]. Viral hepatitis was also considered as a factor associated with higher ALT activity [35], [39]. However, no study had excluded subjects with viral hepatitis at baseline, which might introduce heterogeneity. Still, it might be partly responsible for the higher heterogeneity observed in the male population (I^2^ = 54.7%, P = 0.110) with a higher susceptibility to hepatitis virus and a higher prevalence of chronic hepatitis viral infection in men [40]–[44].

Notably, participants with NAFLD considered as a manifestation of hepatic MetS [45] were not specifically excluded in all of the enrolled studies, which might cause bias and an overestimation for the risk of MetS incidence. However, we speculate the effect was limited for high correlation between the prevalence of NAFLD and MetS in a previous study [46]. Subjects with MetS measured at baseline had been excluded in all of the enrolled studies, which meant the simultaneous exclusion of a majority of subjects with NAFLD. Otherwise, ALT is an available tool in detecting the occurrence and severity of NAFLD in epidemiological survey 9,47. A number of published results from cross-sectional studies have emphasized the major cause of ALT elevation in the general population is NAFLD [35], [48], [49]. To some extent, ALT activity is a tool for representing NAFLD [33] as a manifestation of hepatic metabolic disorder [45], [50]. The impact of NAFLD on MetS incidence can be represented as an ALT-incident MetS association.

Some deficiencies in the enrollment of participants were observed. However, the impact was limited, and the evaluation of an association between ALT elevation and MetS incidence was demonstrated through prospective studies. Better-designed studies, considering comprehensive confounders during enrollment of study participants, are needed to substantiate our results in the future.

The dose-response risk of MetS incidence caused by 5 U/l of ALT elevation was significant in overall and gender-specific evaluations after adjusting for GGT level as another potential liver enzyme also associated with incident MetS [51] (RR: 1.14, 95% CI: 1.11–1.17 in overall population; RR: 1.13, 95% CI: 1.10–1.16 in males, RR: 1.38, 95% CI: 1.20–1.55 in females; all of the enrolled gender-specific RRs were evaluated after adjusting for GGT level; Figure 5). GGT was considered a better risk indicator of MetS incidence due to the significant and consistent GGT-MetS association in previous studies [11], [13], [17]. Through meta-analysis, we found that ALT elevation was associated with higher incidence of MetS independent of GGT levels. In contrast, the RR of incident MetS with 5 U/l of GGT increment lost its significance after adjusting for ALT activity as a confounding covariate in the female population [51]. As a supplement to GGT activity, ALT might be an effective biomarker in the surveillance of MetS incidence, especially in the female population. For other potential confounding variables, no significant heterogeneity was found between subgroups stratified by mean age, ethnicity, follow-up duration, sample size of study population, number of adjusted covariates and adjustment for alcohol intake as a covariate (Figure 5). It suggests the limited effects of these potential factors on the association between ALT activity and MetS incidence.

The mechanism behind the association between increased ALT activities and incident MetS are still not fully elucidated. Elevated ALT activity has been associated with visceral fat accumulation [52]–[55] leading to liver steatosis as a mediator of MetS [56]. Increased visceral fat content, in turn, has been associated with defects in the insulin suppression of glucose production leading to more severe features of insulin resistance including hyperglycemia, hypertriglyceridemia, lower HDL-C concentration, and higher systolic blood pressure as individual MetS component [57], [58]. In subsidiary analysis on ALT activity and incident MetS components, a significant ALT-hyperglycemia association indicates insulin resistance, and subsequent increased hepatic gluconeogenesis, have a crucial role in the association of ALT activity with MetS incidence. Meanwhile, gender disparity exists in the pathophysiology of MetS [59]. Females are intrinsically more prone to insulin resistance, as impaired glucose homeostasis has been linked to X-chromosomal loci [60]–[63]. In support, our results confirm that women are more susceptive to MetS occurrence to the same extent of ALT elevation. In addition, insulin resistance and the ensuing MetS caused by ALT elevation are considered a crucial link in ALT-CVD incidence [33], [64], and our results confirm this central link through meta-analysis. However, these speculations need confirmation in future mechanistic investigations.

Our findings are highly applicable to clinical practice. All the enrolled studies adopt internationally-recognized definition (NCEP-ATP-III [29] or IDF criteria [30]) designed to aid clinicians in recognizing MetS. Sensitivity analysis showed no significant change from omitting any single study, enhancing the reliability of our results. The strength of the association between ALT activity and incident MetS is now even clearer through this meta-analysis. It will provide clinicians more convincing evidence when counseling subjects during health check-ups. In addition, it indicates that gender should be considered when evaluating the ALT-MetS association.

Besides the previous defects referred, several limitations in this meta-analysis should be addressed. First, the average age of the enrolled participants exceeded 40 years in eight data clusters (out of nine data clusters overall). No prospective research was performed in younger adults, adolescents, and children. Further studies should be conducted in these groups on increasing prevalence of MetS [65], [66]. Second, this is not a mechanistic study. The biology underlying the association between ALT elevation and incident MetS deserves further investigation through new perspectives, such as metabolomics [67]. Third, in addition to the intrinsic difference between ALT measurement assays in different institutes, heterogeneity was observed in our meta-analysis due to many factors, including differences in participant enrollments, statistics used for comparison, follow-up lengths, and adjusted covariates. Even the subgroup analysis conducted can not explain all the sources of heterogeneity.

In conclusion, this meta-analysis of prospective cohort studies provides evidence that ALT elevation is consistently and independently associated with MetS occurrence, especially in the female population. The ALT assay therefore has the potential to serve as a valuable tool for assessing the risk of MetS incidence. More prospective studies are needed in different ethnicities to confirm this association and mechanism studies are warranted in the future.

## Supporting Information

Figure S1 Relative risks of incident MetS per SD increment of logALT. Abbreviations: SD: standard deviation.
(TIF)

Table S1 Quality assessment of the studies included in the meta-analysis by NOS*.
(DOCX)

Checklist S1 PRISMA Checklist.
(DOC)

Flow Diagram S1 (DOC)
