# Supplementary figures and images for: Elevated Alanine Aminotransferase Is Strongly Associated with Incident Metabolic Syndrome: A Meta-Analysis of Prospective Studies
Source: PLoS One. 2013 Dec 4;8(12):e80596. doi: 10.1371/journal.pone.0080596 (PMC3851461; doi:10.1371/journal.pone.0080596)

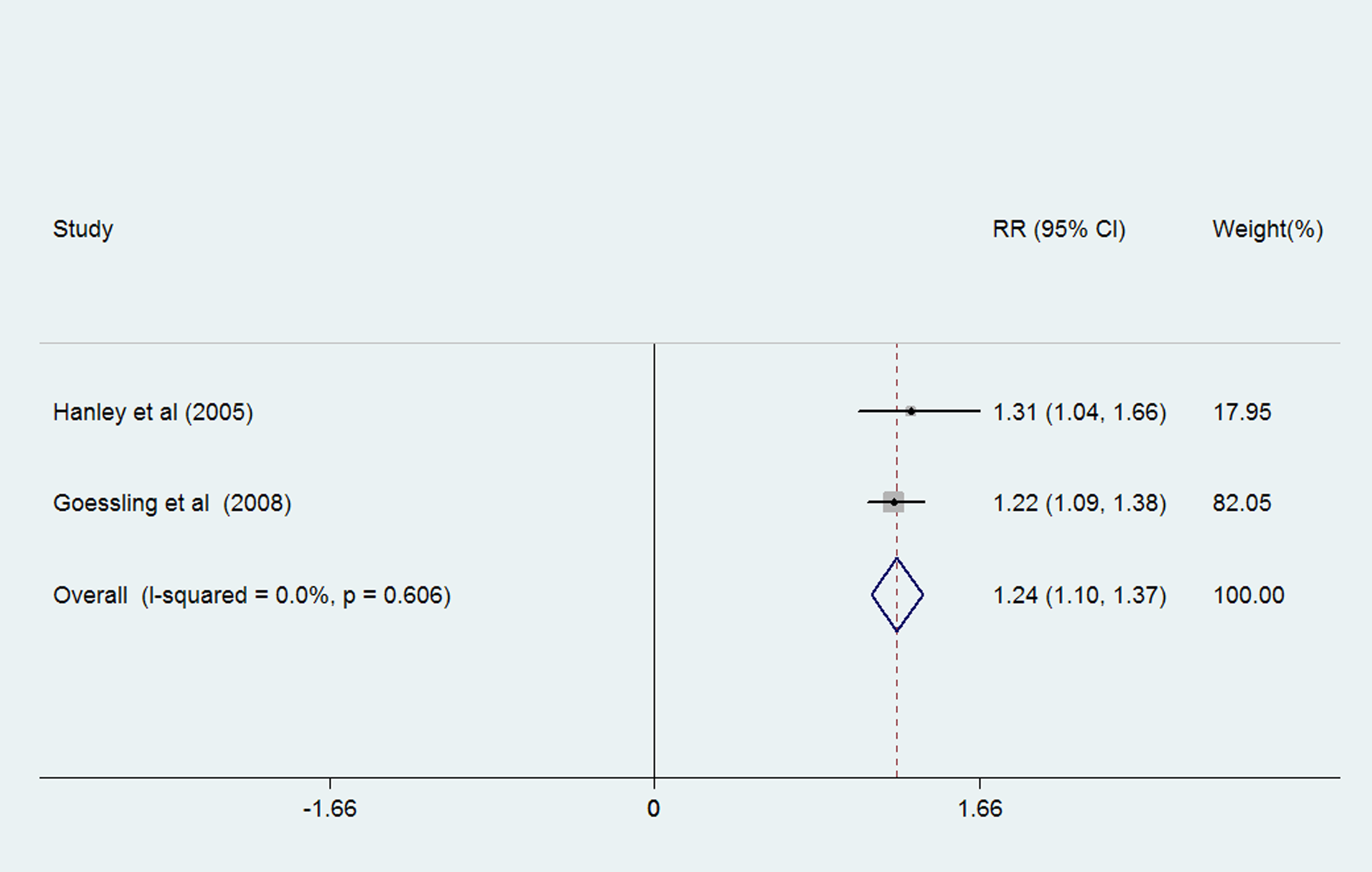

Supplement: Figure S1 — Relative risks of incident MetS per SD increment of logALT. Abbreviations: SD: standard deviation. (TIF) [file pone.0080596.s001.tif]
