# Supplementary material for: Elevated Alanine Aminotransferase Is Strongly Associated with Incident Metabolic Syndrome: A Meta-Analysis of Prospective Studies
Source: PLoS One. 2013 Dec 4;8(12):e80596. doi: 10.1371/journal.pone.0080596 (PMC3851461; doi:10.1371/journal.pone.0080596)
Supplement: Table S1 — Quality assessment of the studies included in the meta-analysis by NOS*. (DOCX) [file pone.0080596.s002.docx]

Table S1 Quality assessment of the studies included in the meta-analysis by NOS*

| NOS scale | NORIYUKI NAKANISHI et al.[11],2004 | Anthony J.G. Hanley et al.[12],2005 | PHILIPPE ANDRE et al.[13], 2007 | R. K. Schindhelm et al.[14], 2007 | Wolfram Goessling et al.[15], 2008 | Sook-Kyoung Jo et al.[16], 2009 | Yu XU et al.[17],  2011 |
| --- | --- | --- | --- | --- | --- | --- | --- |
| **A Selection (maximum 4)**  **1.Representativeness of general community population**  **2.Subjects with ALT elevation were drawn from the same community with participants within normal range**  **3.Ascertainment the exposure of high ALT activity**  **4.MetS was not present at baseline**  **B Comparability (maximum 2)**  **5.Controlled for age and gender**  **6.Controlled for 2 or more variables including alcohol intake, cigarette smoking, family history**  **C Outcome (maximum 3)**  **7. Clearly defined MetS outcome by certain criteria**  **8. Adequate duration of follow-up (>5 years)**  **9. Adequacy of follow-up rate (>90%) of cohorts**    **Total scores (maximum 9)** | 1  1  1  1  1  1  1  1  1  9 | 1  1  1  1  1  0  1  1  0  7 | 1  1  1  1  1  1  1  0  0  7 | 1  1  1  1  1  0  1  1  1  8 | 1  1  0  1  1  1  1  1  1  8 | 1  1  1  1  1  0  1  0  0  6 | 1  1  1  1  1  1  1  0  1  8 |

* NOS: Newcastle-Ottawa Scale

“1” meant the study was correspond to the NOS criteria,” 0” meant the study wasn’t correspond to the NOS criteria
