# Supplementary material for: Elevated Alanine Aminotransferase Is Strongly Associated with Incident Metabolic Syndrome: A Meta-Analysis of Prospective Studies
Source: PLoS One. 2013 Dec 4;8(12):e80596. doi: 10.1371/journal.pone.0080596 (PMC3851461; doi:10.1371/journal.pone.0080596)
Supplement: Flow Diagram S1 — (DOC) [file pone.0080596.s004.doc]

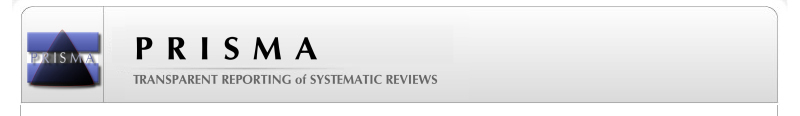
**PRISMA 2009 Flow Diagram**

**Screening**

**Included**

**Eligibility**

**Identification**

Records identified through database searching
(n = 3747 )

Additional records identified through other sources
(n = 0 )

Records after duplicates removed
(n = 1805 )

Records screened
(n = 1942 )

Records excluded
(n = 1918 )

Full-text articles assessed for eligibility
(n = 24 )

Full-text articles excluded, with reasons
(n = 14 )

Studies included in qualitative synthesis
(n = 10 )

Studies included in quantitative synthesis (meta-analysis)
(n = 7 )
